# Supplementary material for: Host-pathogen interactions in the Plasmodium-infected mouse liver at spatial and single-cell resolution
Source: Nat Commun. 2024 Aug 19;15:7105. doi: 10.1038/s41467-024-51418-2 (PMC11333755; doi:10.1038/s41467-024-51418-2)
Supplement: Supplementary file 3 — Description of Additional Supplementary Files [file 41467_2024_51418_MOESM3_ESM.pdf]

Description of Additional Supplementary Files:

Supplementary Data 1: Differential gene expression analysis results (Wilcoxon-rank-sum test) between identified clusters of Spatial Transcriptomics (ST) data (sheet1), Visium data (sheet 2) and Shared data, which contains differentially expressed genes shared between the ST and Visium data. Only significant genes (p <= 0.05, Bonferroni corrected) are shown.

Supplementary Data 2: Differential gene expression results between timepoints of ST data showing significantly upregulated genes (p <= 0.05, Bonferroni corrected) at 12 hours post infection (hpi) (sheet 1), 24hpi (sheet 2) and 38hpi (sheet 3).

Supplementary Data 3: Correlation coefficients between gene expression and distance to the parasites (within 400 μm of the parasite neighborhood) sorted by increasing correlation for either all genes (*P. berghei* and the murine host) or only considering genes of the murine host alone. Only significant genes (p <= 0.05, Bonferroni corrected) were considered.

Supplementary Data 4: Differential gene expression of snRNA-seq data, defining cell type signatures (sheet 1) and showing differential gene expression patterns of cell type markers of immune cell subpopulations (B cells (sheet 2), monocytes and dendritic cells (sheet 2), Kupffer cells (sheet 3) and T & NK cells (sheet 4)) between different infection conditions (12, 24 and 38 hpi/SGC).
