# Supplementary material for: Host-pathogen interactions in the Plasmodium-infected mouse liver at spatial and single-cell resolution
Source: Nat Commun. 2024 Aug 19;15:7105. doi: 10.1038/s41467-024-51418-2 (PMC11333755; doi:10.1038/s41467-024-51418-2)
Supplement: Supplementary file 8 — Reporting Summary [file 41467_2024_51418_MOESM8_ESM.pdf]

nature portfolio

Corresponding author(s): Johan Ankarklev, Franziska Hildebrandt

Last updated by author(s): Jul 9, 2024

Reporting Summary

Nature Portfolio wishes to improve the reproducibility of the work that we publish. This form provides structure for consistency and transparency in reporting. For further information on Nature Portfolio policies, see our Editorial Policies and the Editorial Policy Checklist.

Statistics

For all statistical analyses, confirm that the following items are present in the figure legend, table legend, main text, or Methods section.

n/a	Confirmed	
☐	☒	The exact sample size (*n*) for each experimental group/condition, given as a discrete number and unit of measurement
☐	☒	A statement on whether measurements were taken from distinct samples or whether the same sample was measured repeatedly
☐	☒	The statistical test(s) used AND whether they are one- or two-sided<br>*Only common tests should be described solely by name; describe more complex techniques in the Methods section.*
☐	☒	A description of all covariates tested
☐	☒	A description of any assumptions or corrections, such as tests of normality and adjustment for multiple comparisons
☐	☒	A full description of the statistical parameters including central tendency (e.g. means) or other basic estimates (e.g. regression coefficient) AND variation (e.g. standard deviation) or associated estimates of uncertainty (e.g. confidence intervals)
☐	☒	For null hypothesis testing, the test statistic (e.g. *F*, *t*, *r*) with confidence intervals, effect sizes, degrees of freedom and *P* value noted<br>*Give P values as exact values whenever suitable.*
☒	☐	For Bayesian analysis, information on the choice of priors and Markov chain Monte Carlo settings
☐	☒	For hierarchical and complex designs, identification of the appropriate level for tests and full reporting of outcomes
☐	☒	Estimates of effect sizes (e.g. Cohen's *d*, Pearson's *r*), indicating how they were calculated

Our web collection on statistics for biologists contains articles on many of the points above.

Software and code

Policy information about availability of computer code

Data collection	Data was collected using Illumina500 and Illumina2000 software including DRAGEN (v3.5.7, v3.6.3 and v3.6.6) to receive .fastq files.
Data analysis	Analysis was performed in R (v.) using publicly available and peer-reviewed analysis pipelines in bash, R and python. These are described in detail in the materials and methods section of the manuscript and listed below:<br><br>stpipeline (v.1.8.1), STAR (v.2.6.1e), spaceranger (v.2.0.0), cellranger (v.3.0.0), R (v 4.0.5), STUtility (v 0.1.0), Seurat (v 4.1.1), harmony (v.0.1.0), gprofiler2 (v.1.0), python (v 2.7.18), seaborn (v.0.12.2), hepaquery (v.0.1), sklearn's (v 0.23.1), DoubletFinder (v 2.0.3), scmap (v.1.16.0), stereoscope (v.0.3.1), R (v4.2.2), Seurat (v.4.3.0)

For manuscripts utilizing custom algorithms or software that are central to the research but not yet described in published literature, software must be made available to editors and reviewers. We strongly encourage code deposition in a community repository (e.g. GitHub). See the Nature Portfolio guidelines for submitting code & software for further information.

Data

Policy information about availability of data

All manuscripts must include a data availability statement. This statement should provide the following information, where applicable:

- Accession codes, unique identifiers, or web links for publicly available datasets
- A description of any restrictions on data availability
- For clinical datasets or third party data, please ensure that the statement adheres to our policy

Data is available on GeneExpression Omnibus (accession numbers: GSE268018, GSE268068 and GSE268112) and processed data is available on zenodo with the doi: https://zenodo.org/records/8386528. Code is available on github (https://github.com/ANKARKLEVLAB/STPbml)

Research involving human participants, their data, or biological material

Policy information about studies with human participants or human data. See also policy information about sex, gender (identity/presentation), and sexual orientation and race, ethnicity and racism.

Reporting on sex and gender	*Use the terms sex (biological attribute) and gender (shaped by social and cultural circumstances) carefully in order to avoid confusing both terms. Indicate if findings apply to only one sex or gender; describe whether sex and gender were considered in study design; whether sex and/or gender was determined based on self-reporting or assigned and methods used.*<br>*Provide in the source data disaggregated sex and gender data, where this information has been collected, and if consent has been obtained for sharing of individual-level data; provide overall numbers in this Reporting Summary. Please state if this information has not been collected.*<br>*Report sex- and gender-based analyses where performed, justify reasons for lack of sex- and gender-based analysis.*
Reporting on race, ethnicity, or other socially relevant groupings	*Please specify the socially constructed or socially relevant categorization variable(s) used in your manuscript and explain why they were used. Please note that such variables should not be used as proxies for other socially constructed/relevant variables (for example, race or ethnicity should not be used as a proxy for socioeconomic status).*<br>*Provide clear definitions of the relevant terms used, how they were provided (by the participants/respondents, the researchers, or third parties), and the method(s) used to classify people into the different categories (e.g. self-report, census or administrative data, social media data, etc.)*<br>*Please provide details about how you controlled for confounding variables in your analyses.*
Population characteristics	*Describe the covariate-relevant population characteristics of the human research participants (e.g. age, genotypic information, past and current diagnosis and treatment categories). If you filled out the behavioural & social sciences study design questions and have nothing to add here, write "See above."*
Recruitment	*Describe how participants were recruited. Outline any potential self-selection bias or other biases that may be present and how these are likely to impact results.*
Ethics oversight	*Identify the organization(s) that approved the study protocol.*

Note that full information on the approval of the study protocol must also be provided in the manuscript.

Field-specific reporting

Please select the one below that is the best fit for your research. If you are not sure, read the appropriate sections before making your selection.

☒ Life sciences ☐ Behavioural & social sciences ☐ Ecological, evolutionary & environmental sciences

For a reference copy of the document with all sections, see nature.com/documents/nr-reporting-summary-flat.pdf

Life sciences study design

All studies must disclose on these points even when the disclosure is negative.

Sample size	No sample-size calculations were performed. Sample size was chosen to include at least three biological replicates of each condition for each experiment while still following the 3R rule for animal experiments in a non-clinical setting.
Data exclusions	Data that did not produce usable sequencing data that did not pass initial quality controls were excluded from the data.
Replication	To ensure experimental reproducibility multiple experiments on the same sample (technical replicate) as well as different samples (biological replicate) were performed. To ensure reproducibility of the code, the code execution was repeated multiple times.
Randomization	Experimental groups were defined by the infection status of the animals and were not randomized. Randomization does not apply to our studies as they were not clinical and defined by infection status.
Blinding	Investigators that manually analyzed the occurence of spatial structures including antibody stainings of cell types were blinded. In addition, computational analyses were performed in an unbiased fashion.

Reporting for specific materials, systems and methods

We require information from authors about some types of materials, experimental systems and methods used in many studies. Here, indicate whether each material, system or method listed is relevant to your study. If you are not sure if a list item applies to your research, read the appropriate section before selecting a response.

Materials & experimental systems

n/a	Involved in the study
☐	☒ Antibodies
☒	☐ Eukaryotic cell lines
☒	☐ Palaeontology and archaeology
☐	☒ Animals and other organisms
☒	☐ Clinical data
☒	☐ Dual use research of concern
☒	☐ Plants

Methods

n/a	Involved in the study
☒	☐ ChIP-seq
☒	☐ Flow cytometry
☒	☐ MRI-based neuroimaging

Antibodies

Antibodies used	Plasmodium berghei UIS4 (Nordic BioSite, cat.no: LS-C204260-400), monoclonal CD4 (Thermo Fisher, cat.no: MA1-146, clone GK1.5), monoclonal CD8 (Thermo Fisher Scientific, cat.no: MA5-29682, clone 208), monoclonal F4/80 (Thermo Fisher Scientific, cat.no: MA5-16624, clone CI:A3-1), monoclonal CD27 (Thermo Fisher Scientific, cat.no: MA5-29671, clone 12), monoclonal CD11b (Thermo Fisher Scientific; cat.no: 53-0112-82, clone M1/70), monoclonal CD11c (Thermo Fisher Scientific, cat.no: 42-0114-82, clone N418), Donkey anti-Rat IgG (H+L) Highly Cross- Adsorbed Secondary Antibody, Alexa FluorTM 488, InvitrogenTM (cat.no: A21208), Donkey anti-Rabbit IgG (H+L) Highly Cross-Adsorbed Secondary Antibody, Alexa Fluor™ 555 (cat.no: A-31572), Donkey anti-Rat IgG (H+L) Highly Cross- Adsorbed Secondary Antibody, Alexa FluorTM 647 (cat.no: A78947), Donkey anti-Rabbit IgG (H+L) Highly Cross-Adsorbed Secondary Antibody, Alexa FluorTM Plus 647 (cat.no: A32795).
Validation	All antibodies are commercially available and have validation data and relevant publications available on their corresponding manufacturers website.

Animals and other research organisms

Policy information about studies involving animals; ARRIVE guidelines recommended for reporting animal research, and Sex and Gender in Research

Laboratory animals	8-9-week old female mice of Mus musculus C57BL/6 were used for the study. The mice were maintained with a 12h day/night light cycle and at ambient temperature and humidity in accordance with NIH guidelines.<br>Anopheles stephensi, maintained at 25-27oC, 80% humidity, and with a 12h day/night light cycle
Wild animals	The study did not involve wild animals
Reporting on sex	Only female mice, which were identified as such by optic evaluation by the responsible at the animal facility were considered in our study. Sex differences were not considered in this study in order to reduce the complexity of the study in order to remove it as a covariate, since we did not consider it to be of main relevance here.
Field-collected samples	The study did not involve samples collected from the field
Ethics oversight	The study was performed in strict accordance with the recommendations from the Guide for Care and Use of Laboratory Animals of the National Institutes of Health (NIH). The animal use was done in accordance with the National Institute of Allergy and Infectious Diseases Animal Care and Use Committees (NIAID ACUC), proposal LMVR 22

Note that full information on the approval of the study protocol must also be provided in the manuscript.

Plants

Seed stocks	*Report on the source of all seed stocks or other plant material used. If applicable, state the seed stock centre and catalogue number. If plant specimens were collected from the field, describe the collection location, date and sampling procedures.*
Novel plant genotypes	*Describe the methods by which all novel plant genotypes were produced. This includes those generated by transgenic approaches, gene editing, chemical/radiation-based mutagenesis and hybridization. For transgenic lines, describe the transformation method, the number of independent lines analyzed and the generation upon which experiments were performed. For gene-edited lines, describe the editor used, the endogenous sequence targeted for editing, the targeting guide RNA sequence (if applicable) and how the editor was applied.*
Authentication	*Describe any authentication procedures for each seed stock used or novel genotype generated. Describe any experiments used to assess the effect of a mutation and, where applicable, how potential secondary effects (e.g. second site T-DNA insertions, mosiacism, off-target gene editing) were examined.*
